# Effects of Bone Morphogenetic Protein‐7 on Steroid‐Induced Extracellular Matrix Accumulation in Human Trabecular Meshwork Cells

**DOI:** 10.1096/fba.2025-00080

**Published:** 2025-05-12

**Authors:** Eun Woo Kim, Jin‐Ok Choi, Min Kyung Chae, Jin‐Sol Lee, Chang Ha Lee, Jo Eun Um, Nam Hee Kim, Jihyeong Kim, Wungrak Choi, Chan Yun Kim

**Affiliations:** ^1^ Department of Ophthalmology, Institute of Vision Research Yonsei University College of Medicine Seoul Republic of Korea; ^2^ Division of AI and Data Analysis Neurogrin Inc. Seoul Republic of Korea; ^3^ MET Life Science Seoul Republic of Korea; ^4^ Oral Cancer Research Institute Yonsei University College of Dentistry Seoul Republic of Korea

**Keywords:** bone morphogenetic protein‐7, extracellular matrix, fibrosis, glaucoma, steroid, trabecular meshwork, transforming growth factor beta

## Abstract

Long‐term steroid use, though essential for treating eye diseases, can cause increased intraocular pressure (IOP) in susceptible individuals and may lead to steroid‐induced glaucoma in a subset of patients. This study investigated the effect of bone morphogenetic protein‐7 (BMP‐7) on steroid‐induced extracellular matrix (ECM) synthesis in human trabecular meshwork (TM) cells. We sought to explore the potential of BMP‐7 as a protective agent against steroid‐induced ECM accumulation in the TM. Human TM cells (HTMCs) were treated with either steroids alone or a combination of steroids and BMP‐7 to compare their effects on ECM production. BMP‐7, known for its transforming growth factor beta (TGF‐β) antagonistic properties, was administered using a micellized protein transduction domain (mPTD)‐fused BMP‐7 polypeptide to enhance activity. Gene expression analysis was conducted to identify specific genes involved in ECM regulation. BMP‐7 effectively inhibited steroid‐induced ECM accumulation in HTMCs. There was a significant reduction in ECM production in the steroid and BMP‐7 co‐treated group compared with that in the steroid‐only group. Furthermore, several genes involved in ECM regulation were identified in the co‐treatment, underscoring BMP‐7's potential role in modulating ECM metabolism. These findings demonstrate that BMP‐7 exerts protective, anti‐fibrotic effects in HTMCs by inhibiting steroid‐induced ECM synthesis. BMP‐7 may serve as a promising therapeutic target for preventing or treating steroid‐induced glaucoma by maintaining normal aqueous humor outflow and preventing IOP elevation.

## Graphical abstract

Long‐term steroid use can elevate intraocular pressure (IOP) and lead to steroid‐induced glaucoma by promoting extracellular matrix (ECM) accumulation in the trabecular meshwork (TM). This study demonstrates that bone morphogenetic protein‐7 (BMP‐7), delivered via a micellized protein transduction domain (mPTD), effectively inhibits steroid‐induced ECM synthesis in human TM cells. BMP‐7 co‐treatment reduced ECM production and modulated ECM‐related gene expression, highlighting its potential as a therapeutic strategy to prevent steroid‐induced glaucoma.

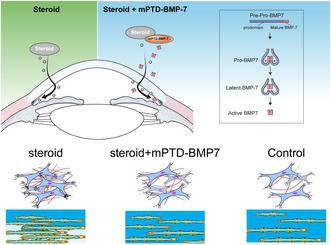

**Abbreviations**
BMP‐7: bone morphogenetic protein‐7
CVF: connective tissue volume fraction
DEGs: differentially expressed genes
ECM: extracellular matrix
GAGs: glycosaminoglycans
GO: gene ontology
HTMCs: human trabecular meshwork cells
IOP: intraocular pressure
KEGG: Kyoto Encyclopedia of Genes and Genomes
mPTD: micellized protein transduction domain
NO: nitric oxide
PBS: phosphate‐buffered saline
RT‐qPCR: reverse transcription‐quantitative PCR
TGF‐β: transforming growth factor beta
TM: trabecular meshwork
TMCM: TM cell medium

## 1 Introduction

Steroids are commonly used in the pharmacological management of various ocular conditions. However, their use is associated with a significant risk of increased intraocular pressure (IOP), which can lead to the development of steroid‐induced glaucoma [1, 2]. This condition may result in optic nerve damage and irreversible vision loss. Although several mechanisms have been proposed, including the well‐established transforming growth factor beta (TGF‐β)/Smad3‐mediated extracellular matrix (ECM) accumulation pathway, the precise and multifactorial processes underlying IOP elevation following steroid administration remain incompletely understood [3, 4, 5]. The degree of IOP elevation varies depending on the dose and method of administration, with notable differences observed among individuals, even when receiving equivalent doses [6]. Notably, not all individuals exposed to steroids develop glaucoma, as the risk depends on genetic factors, steroid dosage, treatment duration, and baseline ocular conditions. Children are particularly susceptible to elevated IOP, necessitating vigilant monitoring and consideration of alternative therapies when possible. In severe cases, surgical intervention may be necessary to regulate IOP and preserve vision, highlighting the need to balance the therapeutic advantages of steroids against their potential adverse effects [5]. Therefore, identifying the etiology of steroid‐induced glaucoma is crucial for ensuring the safe administration of steroids.

The most widely accepted mechanism underlying steroid‐induced elevation of IOP involves the activation of ECM synthesis in the trabecular meshwork (TM), which is the primary route for aqueous humor outflow [7, 8]. Previous studies have demonstrated increased levels of TGF‐β in the aqueous humor of individuals with steroid‐induced glaucoma [1, 9, 10, 11]. Steroids can enhance the expression of TGF‐β, which in turn accelerates the accumulation and remodeling of ECM components within the TM. Excess ECM obstructs pores in the TM that normally facilitate the drainage of aqueous humor into the venous system, leading to compromised drainage and increased IOP [7]. Prolonged IOP elevation can lead to optic nerve damage, culminating in the development of glaucoma.

Bone morphogenetic protein‐7 (BMP‐7), a member of the TGF‐β superfamily, plays a crucial role in the development of bone and cartilage and exhibits regenerative properties in various tissues. BMP‐7 functions as an antagonist of TGF‐β by inhibiting TGF‐β‐associated signaling pathways [12]. This inhibition is expected to prevent excessive deposition and remodeling of the ECM, which contributes to elevated IOP in steroid‐induced glaucoma. Owing to its antagonistic relationship with TGF‐β, BMP‐7 has emerged as a promising therapeutic candidate, with the potential to alleviate fibrotic changes in the TM, restore normal aqueous humor outflow, and reduce the risk of glaucoma.

Given that TGF‐β is involved in steroid‐induced ECM accumulation and that BMP‐7 inhibits TGF‐β, we explored the potential of BMP‐7 in preventing steroid‐induced ECM accumulation. To enhance the activity of BMP‐7, we utilized a micellized protein transduction domain (PTD)‐fused BMP‐7 polypeptide (mPTD‐BMP‐7). This modified form of BMP‐7 is designed to penetrate cells more efficiently and exert a stronger inhibitory effect on TGF‐β signaling [13, 14, 15]. This study aimed to investigate the proteins affected by steroids and BMP‐7 in human TM cells (HTMCs) and to identify associated gene expression changes using RNA‐sequencing.

## 2 Materials and Methods

### 2.1 Animals

All animal experiments were conducted in accordance with ethical guidelines and regulations for experimental research and were approved by the Institutional Animal Care and Use Committee of Korea Conformity Laboratories (approval number: KCL‐IACUC‐2023‐0116). Specific pathogen‐free New Zealand White Rabbits (DooYeol Biotech, Seoul, Korea) were used for in vivo pharmacokinetic analysis and safety assessment. The animals were housed under controlled conditions (temperature: 20°C–26°C; humidity: 40%–60%). A total of 12 New Zealand White rabbits, each weighing between 2.5 and 3 kg, were used in this study, in compliance with the guidelines set by the Association for Research in Vision and Ophthalmology. Approval was obtained from the Institutional Animal Ethics Committee.

### 2.2 mPTD‐BMP‐7 Preparation

The bacterial expression vector for mPTD‐BMP‐7 and protein purification has been described previously [13, 14, 15, 16, 17]. This PTD fusion peptide enables the direct transduction of proteins across the cell membrane. The denatured polypeptide was micellized with filtered 0.1% egg lecithin (BOC Sciences, Shirley, NY, USA) using sonication. mPTD‐BMP‐7 is a laboratory‐purified protein and the molecular weight of mPTD‐BMP‐7 was shown at approximately 50 kDa location. Assessed by using a particle size analyzer (ELSZ‐2000ZS, Otsuka Electronics), micellization resulted in a homogeneous and stable nanosized particle distribution (Figure S5).

### 2.3 Steroid and mPTD‐BMP‐7 Treatment of Rabbits

The rabbits were divided into three groups, with four rabbits in each group. The right eye of each rabbit was injected, whereas the left eye remained untreated. The control group received balanced salt solution, the second group received dexamethasone (5 mg/mL) injections, and the third group received a combination of dexamethasone and mPTD‐BMP‐7 (20 ng/mL). The rabbits were anesthetized by intramuscular administration of alfaxalone (3–5 mg/kg), followed by local anesthesia using 0.5% proparacaine hydrochloride eye drops. The injections were delivered through three routes into the eye: 0.05 mL into the anterior chamber, 0.05 mL into the vitreous cavity, and 0.2 mL into the subconjunctival space, using a 30‐gauge needle syringe. The injections were administered weekly for 4 weeks, followed by a 4‐week observation period without additional treatment. After 8 weeks, the rabbits were euthanized, and their eyes were collected for histological analysis.

### 2.4 Histological Examination

After sacrificing the animals, the eyeballs were promptly removed and cut into small 1 mm^3^ clumps. The samples were fixed by immersion in 2.5% glutaraldehyde (16220; Electron Microscopy Sciences) diluted in 0.1 M phosphate buffer for 2 h at room temperature. Samples were rinsed twice with 0.1 M phosphate buffer and post‐fixed with 1% osmium tetroxide (75632; Sigma‐Aldrich) diluted in 0.1 M phosphate buffer for 1 h at 4°C. Next, the samples were dehydrated with a series of graded ethyl alcohol solutions (100983; Merck Millipore) and followed by acetone (A18‐4; Fisher Scientific). The samples were then embedded in EPON 812. Ultrathin sections (70–80 nm) were obtained using an ultramicrotome (Leica Ultracut UCT; Leica, Wetzlar, Germany), stained with uranyl acetate (NC1375332; Fisher Scientific) and lead citrate (NC1588038; Fisher Scientific), and examined using a transmission electron microscope (JEM‐1010; JEOL, Tokyo, Japan) at 60 kV. Sections from each eye, extending from the corneal apex to the optic nerve plane, were subjected to Masson's trichrome staining to assess collagen expression. Images were captured using a Leica DM4 microscope (DM400B; Leica, Wetzlar, Germany) and analyzed using the ImageJ software. Changes in TM cells were quantified by measuring the red‐stained area, while TM cell size was calculated as the percentage of red‐stained tissue within the total area. ECM expression was quantified by connective tissue volume fraction (CVF), calculated as the percentage of the blue‐stained tissue area within the total area.

### 2.5 Culture of HTMCs

Primary HTMCs (6950; Sciencell) were utilized for experiments at passages 3–6. The HTMCs were cultured in TM cell medium (TMCM; 6951; Sciencell). All cultures were maintained at 37°C in a humidified incubator with 5% CO_2_ and 95% air.

### 2.6 Treating HTMCs With Steroids and mPTD‐BMP‐7

Two sets of experiments were conducted to investigate ECM mechanics following dexamethasone treatment in HTMCs. In the first experiment, ECM mechanics were observed after treatment with dexamethasone alone, while in the second experiment, ECM mechanics were evaluated over time following treatment with both dexamethasone and mPTD‐BMP‐7. To assess the effect of dexamethasone on HTMCs, cells were cultured in 6‐well plates (5000–10,000/cm^2^) with 2 mL of culture medium per well. One group served as the untreated control, whereas the other two groups were treated with 100 nM dexamethasone (1.6 μL/well) for 1, 2, 3, and 7 days. Additionally, the last group was treated under the same conditions as the dexamethasone‐treated groups but received an additional 20 ng/mL mPTD‐BMP‐7 (1 μL/well). In summary, primary HTMCs were treated with control, dexamethasone, or dexamethasone+mPTD‐BMP‐7 in TMCM for 1, 2, 3, and 7 days. We conducted preliminary experiments using 5, 10, 20, 50, and 100 ng/mL concentrations of mPTD‐BMP‐7. Among these, 20 ng/mL produced the most consistent ECM‐inhibitory effect without cytotoxicity. Cytotoxicity testing results are included in Figure S2.

### 2.7 Imaging the ECM

To visualize F‐actin and fibronectin in the cytoplasm of HTMCs, both treated and control cells were cultured on 24‐well plates with coverslips. Cells were fixed with 4% formaldehyde and permeabilized with 0.1% Triton X‐100. After blocking with 1% bovine serum albumin, cells were incubated with primary antibodies diluted in a blocking buffer at 4°C overnight, followed by incubation with fluorochrome‐conjugated secondary antibodies. Fluorescence was visualized using a confocal microscope (LSM 700; Zeiss). The reagents used for immunofluorescence are as follows: Alexa Fluor 488 phalloidin (1:1000 dilution; A12379; Thermo Fisher Scientific) and fibronectin (1:250 dilution; sc‐8422; Santa Cruz Biotechnology).

### 2.8 Protein Extraction and Western Blot Analysis

Following dexamethasone and mPTD‐BMP‐7 treatment, cells were lysed at the end of each time point (Days 3 and 7), and proteins were extracted for Western blotting. Cells were washed with cold 1× phosphate‐buffered saline (PBS) and harvested by centrifugation at 1000 rpm for 5 min. The resulting cell pellet was lysed in 50 μL RIPA Buffer (Biosesang, Korea) for protein extraction. After centrifugation at 15,000 rpm for 20 min, the lysates were transferred to a new microcentrifuge tube for protein quantification using the BCA assay (Thermo Fisher Scientific, USA). Equal amounts of protein were mixed with 5× sample buffer (ELPIS, Korea) and boiled at 100°C for 10 min. The samples were then loaded onto an 8% sodium dodecyl sulfate‐polyacrylamide gel for electrophoresis, followed by transfer onto a polyvinylidene difluoride membrane (Millipore, USA).

The membrane was blocked with 5% skim milk for 1 h and incubated with the primary antibodies overnight at 4°C. After three washes with 1× TBST, the membrane was incubated with the secondary antibody for 1 h. After three washes with 1× TBST, the protein bands were visualized using an enhanced chemiluminescence detection reagent (Bio‐Rad Laboratories, USA). The antibodies used for Western blotting are listed in Table S1.

Alterations in ECM structure correlated with changes in protein expression, as confirmed by Western blotting. The experiment was repeated three times, and ImageJ software was used for protein quantification.

### 2.9 RNA Extraction and Quantitative Reverse Transcription Polymerase Chain Reaction

HTMCs were seeded in a 6‐well culture dish at a density of 4 × 10^5^ cells/well and cultured for 16 h. The cells were treated with dexamethasone and/or mPTD‐BMP‐7 as previously mentioned. After treatment, the cells were collected for RNA extraction. The supernatant was removed, and the cells were washed once with cold 1× PBS. TRI Reagent (Molecular Research Center, USA) was added to each well (500 μL/well), and the contents were transferred to a microcentrifuge tube. Then, 200 μL of chloroform (Daegung, Korea) was added to each tube. The mixture was vortexed and incubated at room temperature for 10 min, followed by centrifugation at 15,000 rpm for 10 min to separate the phases. The upper aqueous phase containing RNA was carefully transferred to a new tube, and an equal volume of isopropanol (Daegung, Korea) was added. Centrifugation was performed at 15,000 rpm for 10 min. The supernatant was discarded, and 500 μL of 70% ethanol (Millipore, USA) was added to wash the RNA pellet. After removing the supernatant, the samples were air‐dried at room temperature for 30 min. The RNA was resuspended in RNase‐free water and quantified.

Next, reverse transcription‐quantitative PCR (RT‐qPCR) was performed to compare mRNA expression levels. cDNA was synthesized using the RNA‐to‐cDNA EcoDry Premix (Oligo dT) from Takara Bio (Mountain View, CA, USA). PCR was performed using the SYBR Premix Ex Taq (Takara Bio) and the StepOnePlus Real‐Time PCR system (Thermo Fisher Scientific, USA). The forward and reverse primers used are summarized in Table S2. Relative gene expression levels were determined using the comparative 2^−ΔΔCT^ method.

### 2.10 RNA‐Sequencing and Analysis

RNA library preparation and sequencing were conducted by LAS Inc. (Gimpo, Korea; http://www.lascience.co.kr/) using the SMARTER Stranded Total RNA‐seq kit‐v2—Pico Input Mammalian (Takara Bio) according to the manufacturer's instructions. This process included the ligation of RNAs with 3' and 5' adapters, followed by reverse transcription into cDNA. PCR was performed with Illumina index primers to differentiate samples collected at various time points post‐injury from both proximal and distal segments. Sequencing was performed on a NextSeq 500 System (Illumina, San Diego, CA) with 75 bp paired‐end reads.

Quality control was performed using FastQC version 0.11.5, and sequencing adapters and low‐quality bases were trimmed using Skewer version 0.2.2. High‐quality reads were aligned to the reference genome using STAR version 2.6. Quantification of gene expression was achieved with Cuffquant in Cufflinks version 2.2.1, and gene expression values were calculated as fragments per kilobase of transcript per million mapped reads. Differential expression analysis among conditions: control (serum‐free) versus steroid and steroid versus steroid+mPTD‐BMP‐7 treatment was performed using Cuffdiff, identifying differentially expressed genes (DEGs) with a fold‐change cutoff of 2 and a *p*‐value cutoff of 0.05. Unsupervised clustering of a selected subset of DEGs was conducted using R scripts. Scatter plots, volcano plots, and comparisons between sample expression profiles were generated.

Functional enrichment analysis was performed using g:Profiler2 version 0.2.0 to investigate the biological roles of DEGs based on Gene Ontology (GO), Kyoto Encyclopedia of Genes and Genomes (KEGG), and other functional enrichment tools. Initially, 30 DEGs were selected from Table 1, to which an additional 7 DEGs were added based on a broader fold‐change range, resulting in a total of 37 DEGs used for STRING analysis (https://www.string‐db.org). Furthermore, 20 additional genes closely associated with these DEGs were integrated, enabling a comprehensive network analysis. This analysis identified 20 new interactions, providing insight into the intricate relationships between proteins and their roles in gene regulation.

**TABLE 1:** Genes identified as upregulated or downregulated after steroid treatment based on RNA‐sequencing.

	Log_2_ (fold‐change)	*p*	Description of the gene
Upregulated gene name			
MAGED4B	4.504	0.026	MAGE family member D4B [Source Symbol;Acc:HGNC:22880]
MCF2L	4.196	0.002	MCF.2 cell line derived transforming sequence like [Source Symbol;Acc:HGNC:14576]
ISY1‐RAB43	3.541	0.030	ISY1‐RAB43 readthrough [Source Symbol;Acc:HGNC:42969]
HBB	3.488	0.005	Hemoglobin subunit alpha 2 [Source Symbol;Acc:HGNC:4827]
HBA2	2.875	0.011	Hemoglobin subunit alpha 2 [Source Symbol;Acc:HGNC:4824]
JMJD7‐PLA2G4B	2.666	0.002	JMJD7‐PLA2G4B readthrough [Source Symbol;Acc:HGNC:34449]
TRIM73	1.993	0.025	Tripartite motif containing 73 [Source Symbol;Acc:HGNC:18162]
S100A14	1.992	0.049	S100 calcium binding protein A14 [Source Symbol;Acc:HGNC:18901]
GUCA1B	1.852	0.019	Guanylate cyclase activator 1B [Source Symbol;Acc:HGNC:4679]
MYCBPAP	1.658	0.026	MYCBP associated protein [Source Symbol;Acc:HGNC:19677]
VAMP8	1.291	0.033	Vesicle associated membrane protein 8 [Source Symbol;Acc:HGNC:12647]
PSG5	1.250	0.016	Pregnancy specific beta‐1‐glycoprotein 5 [Source Symbol;Acc:HGNC:9522]
TMEM121	1.207	0.033	Transmembrane protein 121 [Source Symbol;Acc:HGNC:20511]
AK9	1.151	0.034	Adenylate kinase 9 [Source Symbol;Acc:HGNC:33814]
Downregulated gene name			
PLA2G4B	−3.215	0.020	Phospholiapase A2 group IVB [Source: HGNC Symbol;Acc:HGNC:9036]
DOCK2	−2.382	0.044	Dedicator of cytokinesis 2 [Source: HGNC Symbol;Acc:HGNC:2988]
FAM220A	−2.095	0.000	Family with sequence similarity 220 member A [Source: HGNC Symbol;Acc:HGNC:22422]
CD36	−2.043	0.007	CD36 molecule [Source: HGNC Symbol;Acc:HGNC:1663]
WSCD1	−1.901	0.012	WSC domain containing 1 [Source: HGNC Symbol;Acc:HGNC:29060]
MRVI1	−1.702	0.004	Murine retrovirus integration site 1 homolog [Source: HGNC Symbol;Acc:HGNC:7237]
FTCDNL1	−1.687	0.024	Formiminotransferase cyclodeaminase N‐terminal like [Source: HGNC Symbol;Acc:HGNC:48661]
MISP3	−1.676	0.007	MISP family member 3 [Source: HGNC Symbol;Acc:HGNC:26963]
IL34	−1.467	0.023	Interleukin 34 [Source: HGNC Symbol;Acc:HGNC:28529]
XKR6	−1.439	0.022	XK related 6 [Source: HGNC Symbol;Acc:HGNC:27806]
SERPINB7	−1.301	0.014	Serpin family B member 7 [Source: HGNC Symbol;Acc:HGNC:13902]
LTK	−1.296	0.027	Leukocyte receptor tyrosine kinase [Source: HGNC Symbol;Acc:HGNC:6721]
CATSPERE	−1.263	0.026	Catsper channel auxiliary subunit epsilon [Source: HGNC Symbol;Acc:HGNC:28491]
KRTAP2‐3	−1.189	0.038	Keratin associated protein 2–3 [Source: HGNC Symbol;Acc:HGNC:18906]
ZC3H11B	−1.079	0.014	Zinc finger CCCH‐type containing 11B [Source: HGNC Symbol;Acc:HGNC:29659]
RAMP1	−1.015	0.034	Receptor activity modifying protein 1 [Source: HGNC Symbol;Acc:HGNC:9843]

### 2.11 Statistical Analysis

All data are presented as the mean ± standard deviation. Differences between groups were analyzed using the Student's *t*‐test and one‐way analysis of variance in SPSS statistical software (version 26.0; IBM Corp., Armonk, NY, USA). *p* < 0.05 were considered statistically significant. All experiments were performed with a minimum of three biological replicates unless otherwise noted.

## 3 Results

### 3.1 Effects of Steroid and mPTD‐BMP‐7 on TGF‐β Levels in Rabbit Aqueous Humor and TM Tissue

**FIGURE 1:**
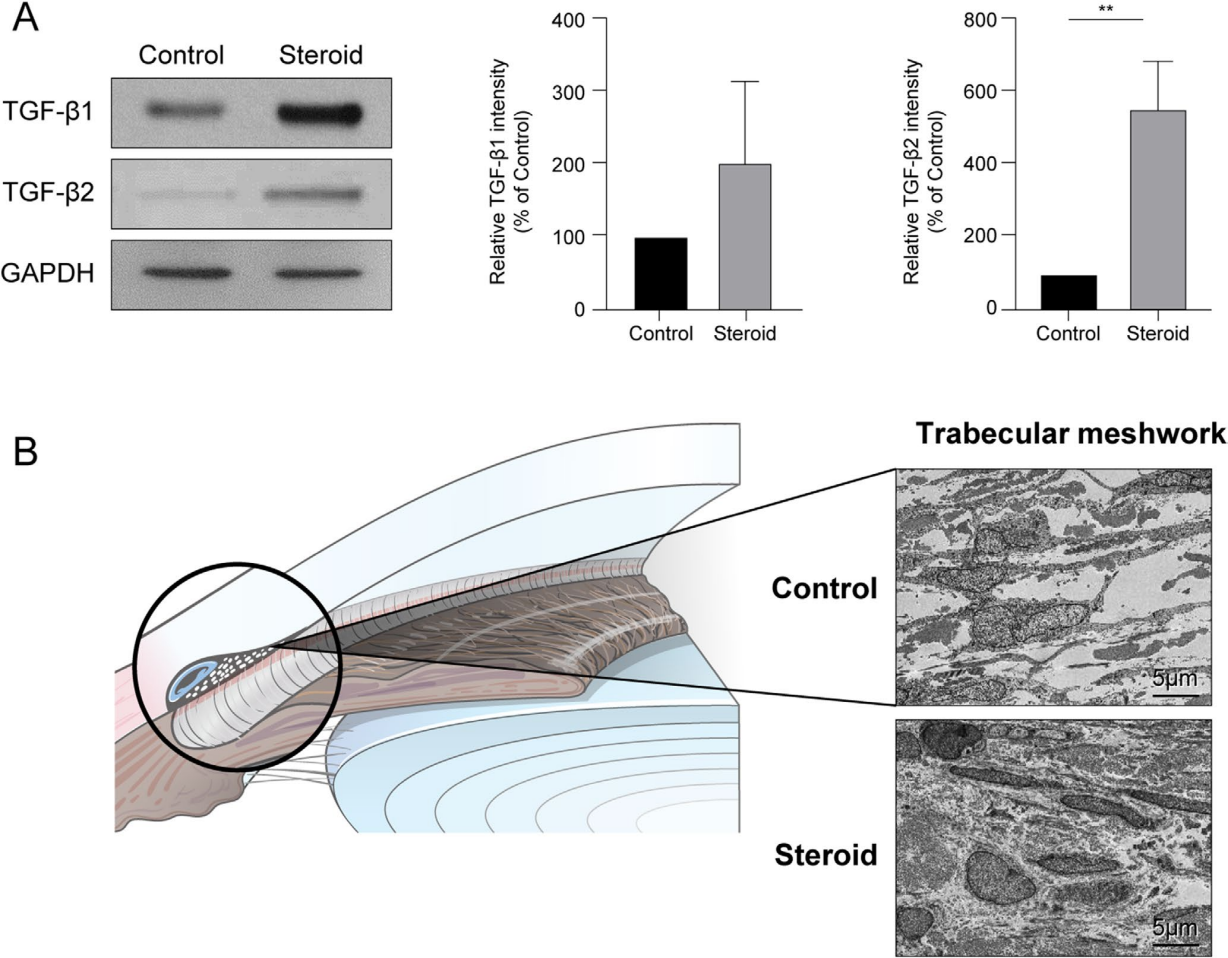
Effect of steroid on transforming growth factor beta (TGF‐β) expression in rabbit trabecular meshwork (TM) cells. (A) The expression level of TGF‐β1 and TGF‐β2 in rabbit aqueous humor before and after dexamethasone administration was assessed via Western blotting. Both TGF‐β1 and TGF‐β2 expression increased in response to dexamethasone. Quantification of the protein bands showed that the increase in TGF‐β2 expression was more pronounced than that in TGF‐β1. (B) Comparison of histological alterations in the TM cells and extracellular matrix (ECM) after dexamethasone injection into the rabbit eye, as observed via transmission electron microscopy (TEM) (scale bar: 5 μm; magnification: 8000×). An increase in the size of TM cells was observed, accompanied by a denser ECM structure (** indicate *p* < 0.05, *p* < 0.01, and *p* < 0.001, respectively). Experiments were performed with a minimum of three biological replicates.

To validate the prevailing theory that TGF‐β concentration in the aqueous humor increases during steroid treatment, the aqueous humor of rabbits administered dexamethasone, a steroid, with or without mPTD‐BMP‐7, was examined. The concentrations of TGF‐β1 and TGF‐β2 in aqueous humor were measured via Western blotting. TGF‐β1 concentration exhibited an approximately two‐fold increase compared to that in the control group, while TGF‐β2 concentration showed a six‐fold increase (Figure 1A). ECM levels are elevated in the TM of patients with steroid‐induced glaucoma. To confirm this finding, we examined histological alterations using transmission electron microscopy (TEM). The results showed that the size of steroid‐treated TM cells increased, and the extracellular space became significantly denser (Figure 1B).

### 3.2 Impact of Steroid and mPTD‐BMP‐7 Treatment on the ECM of HTMCs

**FIGURE 2:**
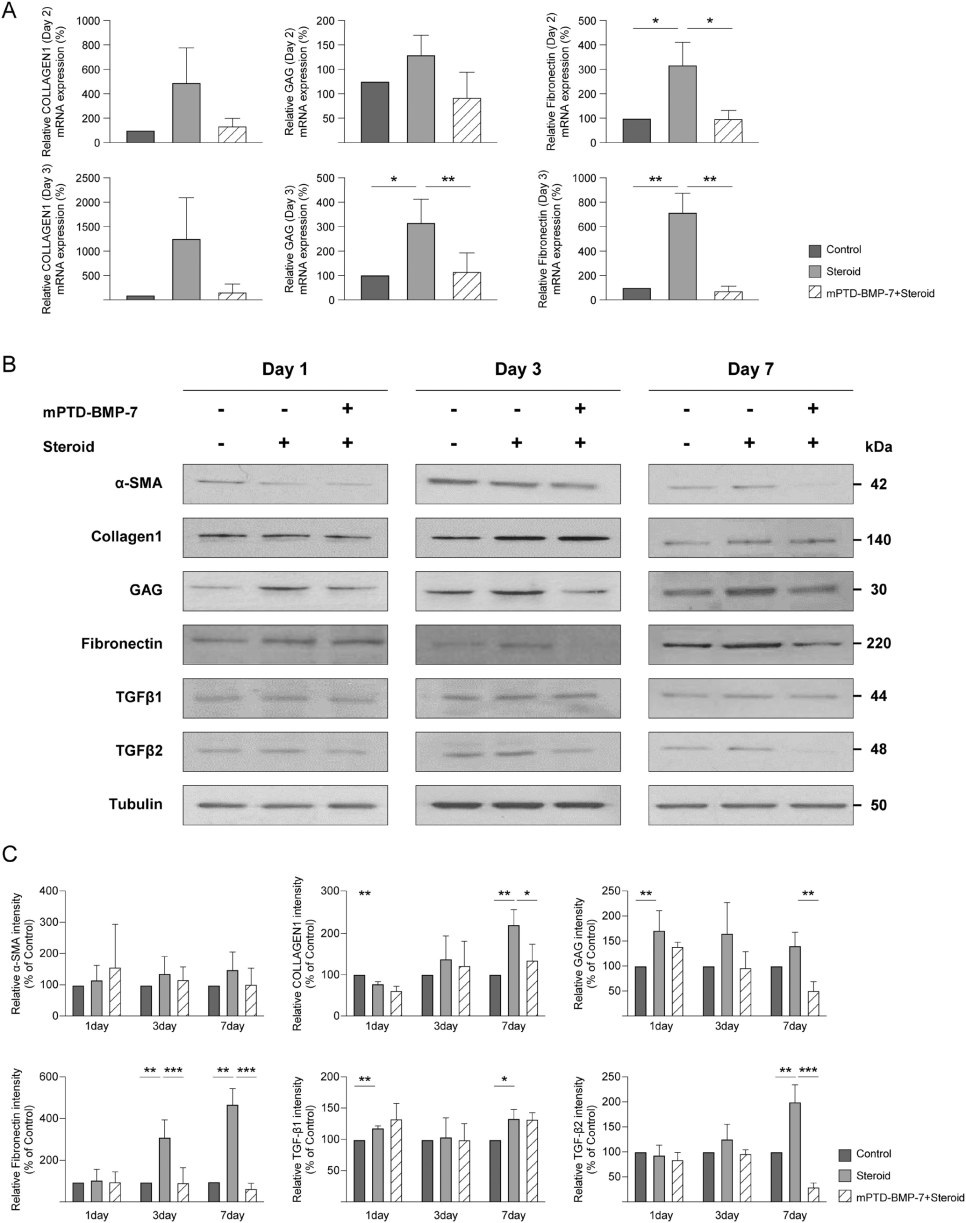
Effect of steroid and micellized protein transduction domain (PTD)‐fused bone morphogenetic protein (BMP‐7) polypeptide (mPTD‐BMP‐7) on ECM in human trabecular meshwork cells (HTMCs). (A) Quantitative reverse transcriptase polymerase chain reaction (RT‐qPCR) for collagen I, glycosaminoglycans (GAGs), and fibronectin in HTMCs treated with 100 nM dexamethasone, with or without 20 ng/mL mPTD‐BMP‐7. mRNA expression levels in HTMCs on Day 2 (upper panels) and Day 3 (lower panels) of treatment. The baseline indicates the HTMCs that are not exposed to dexamethasone or mPTD‐BMP‐7. The relative collagen I, GAG, and fibronectin mRNA expression levels significantly decreased after 3 days of dexamethasone treatment (compared to the controls and after 2 days). This decrease was suppressed by mPTD‐BMP‐7 addition. (B and C) Western blotting for α‐smooth muscle actin, collagen I, GAGs, fibronectin, TGF‐β1, and TGF‐β2 (tubulin was used as the loading control) in HTMCs over the course of 7 days. The representative protein bands on Days 1, 3, and 7 are shown in (B) and with quantification presented in (C). Collagen I, fibronectin, and TGF‐β2 were upregulated in HTMCs exposed to dexamethasone on Day 7, while collagen I, GAGs, fibronectin, and TGF‐β2 were downregulated in HTMCs exposed to dexamethasone along with mPTD‐BMP‐7. (*, **, and *** indicate *p* < 0.05, *p* < 0.01, and *p* < 0.001, respectively). Experiments were performed with a minimum of three biological replicates.

We evaluated the mRNA expression of ECM components [collagen I, glycosaminoglycans (GAGs), and fibronectin] in HTMCs upon steroid treatment via RT‐qPCR. On Day 2, there was a significant increase in fibronectin expression in the cells treated with dexamethasone when compared to that in the control and combination treatment (steroid+mPTD‐BMP‐7) groups. By Day 3, fibronectin levels remained high in the dexamethasone group but decreased in the combination treatment group. The expression of GAGs showed a similar pattern (Figure 2A). Western blotting of the same samples indicated that dexamethasone alone led to an increase in the expression of α‐smooth muscle actin, collagen I, GAGs, fibronectin, TGF‐β1, and TGF‐β2 over time, reaching a peak at Day 7. Combination treatment notably decreased the expression of these proteins compared to dexamethasone treatment alone, indicating that mPTD‐BMP‐7 alleviated the dexamethasone‐induced upregulation of ECM components. Tubulin levels remained consistent across all groups and served as a loading control (Figure 2B).

In the dexamethasone‐treated group, fibronectin and collagen I increased over time, with significant increases observed on Day 7. In contrast, the combination of BMP‐7 and steroid treatment effectively diminished ECM accumulation, as evidenced by the reduced levels of fibronectin and collagen I by Day 7, which approached those in the control group. This finding suggests that BMP‐7 may counteract steroid‐induced ECM accumulation. TGF‐β1 exhibited a modest increase following steroid treatment; however, no significant change was noted when BMP‐7 was co‐administered. In contrast, TGF‐β2 levels showed a marked increase on Day 7 following steroid treatment, while the combination of BMP‐7 and steroid treatment significantly decreased TGF‐β2 expression by Day 7, indicating a pronounced regulatory effect of BMP‐7 (Figure 2C).

### 3.3 Alterations in ECM Volume Upon Steroid and mPTD‐BMP‐7 Co‐Treatment in HTMCs

**FIGURE 3:**
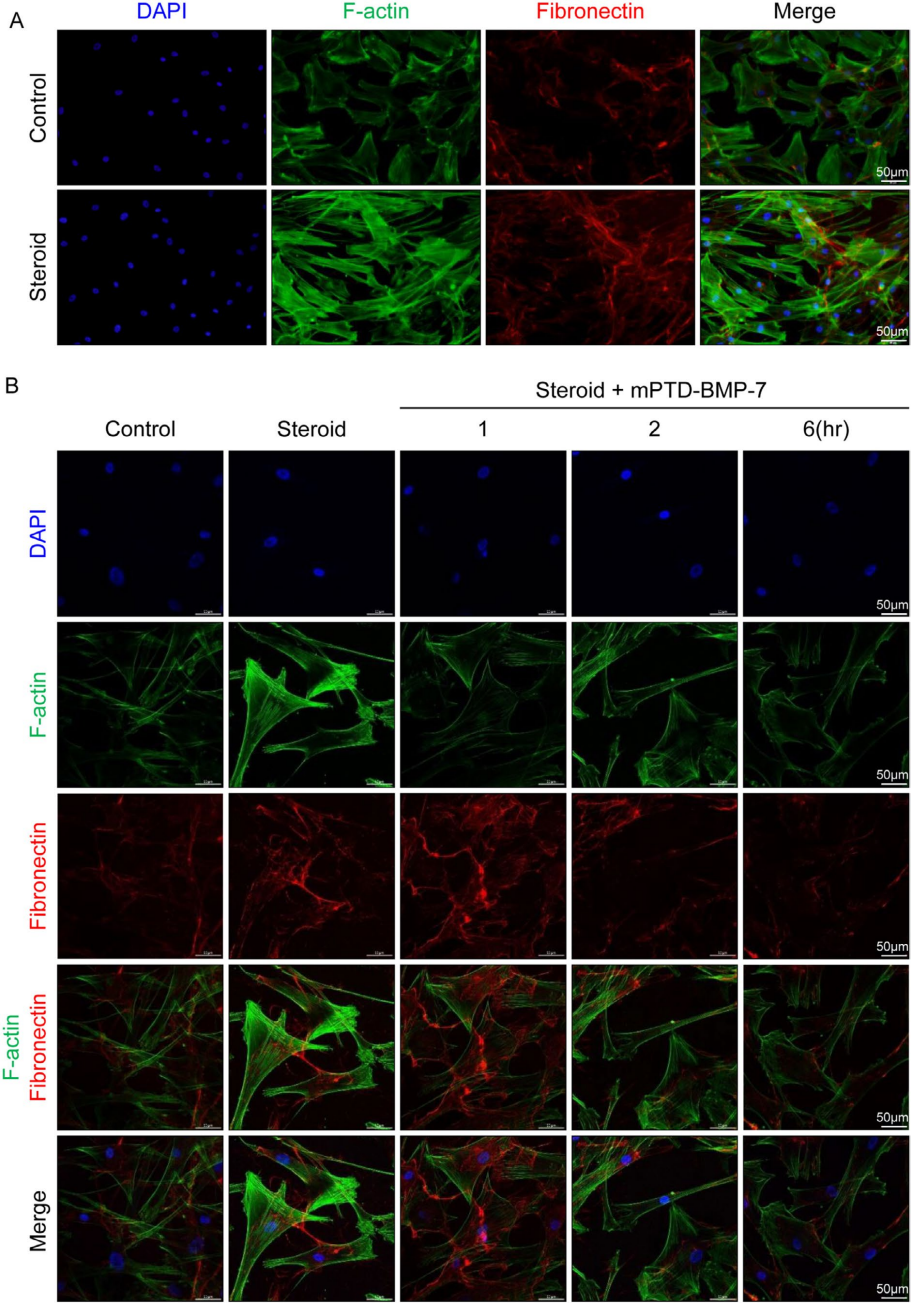
Alterations in ECM components after steroid and mPTD‐BMP‐7 treatment in HTMCs by immunofluorescence staining. mPTD‐BMP‐7 was pre‐treated prior to standard steroid exposure, and the outcomes were evaluated 7 days later. (A) Immunofluorescence detection of F‐actin and fibronectin in HTMCs. The first row shows cells that were not treated with dexamethasone (control group). The second row shows cells treated with dexamethasone. Cells stained with the nuclear stain DAPI are shown in the first column, those stained with F‐actin in the second column, fibronectin in the third column, and a merge of all three in the last column (DAPI+F‐actin+fibronectin). (B) The first row shows cells stained with DAPI, the second shows F‐actin, the third shows fibronectin, the fourth shows F‐actin and fibronectin, and the last row shows a merge of all three. The first column represents no treatment (control), the second indicates cells immediately after dexamethasone and mPTD‐BMP‐7 co‐treatment, and the other three columns represent 1, 2, and 6 h after co‐treatment, respectively. Confocal microscopy images (scale bar: 50 μm; magnification: 400×) reveal that fibronectin levels, which increased with steroid treatment alone, gradually decreased over time following co‐treatment with mPTD‐BMP‐7. Experiments were performed with a minimum of three biological replicates.

We investigated alterations in ECM volume through imaging techniques, focusing on fibronectin, which showed significant variations in prior Western blot analyses, and F‐actin, which forms the structural framework of the ECM. Immunofluorescence staining was utilized to visualize changes in F‐actin and fibronectin within HTMCs before and after dexamethasone administration. DAPI (blue) was utilized to visualize the nucleus, thereby indicating cell positioning and density. The F‐actin that constitutes the cytoskeleton is shown in green, while red denotes fibronectin, an ECM protein. The merged staining images provide a comprehensive overview of the structural organization and protein distribution within the cells (Figure 3A).

In a separate experiment, alterations in ECM volume within HTMCs were investigated following varying durations of exposure to BMP‐7. Five distinct groups were analyzed: a control group (no exposure to steroids or BMP‐7), a steroid‐only group, and three groups that were treated with steroids in conjunction with BMP‐7 exposure for different time intervals (1, 2, or 6 h). The levels of DAPI, F‐actin, and fibronectin were compared across these groups. The control group exhibited the baseline distribution of F‐actin and fibronectin, whereas the steroid‐only group demonstrated a significant increase in the levels of both proteins. Conversely, the groups that were initially exposed to BMP‐7 showed a progressive decline in F‐actin and fibronectin levels, with the reductions becoming increasingly pronounced with the duration of BMP‐7 exposure. The most substantial decrease was noted in the group subjected to BMP‐7 for 6 h. The composite images presented in the bottom row illustrate the correlation between the duration of BMP‐7 exposure and the degree of ECM remodeling observed throughout the week (Figure 3B).

### 3.4 Effect of Steroid and mPTD‐BMP‐7 Co‐Treatment on Rabbit TM

**FIGURE 4:**
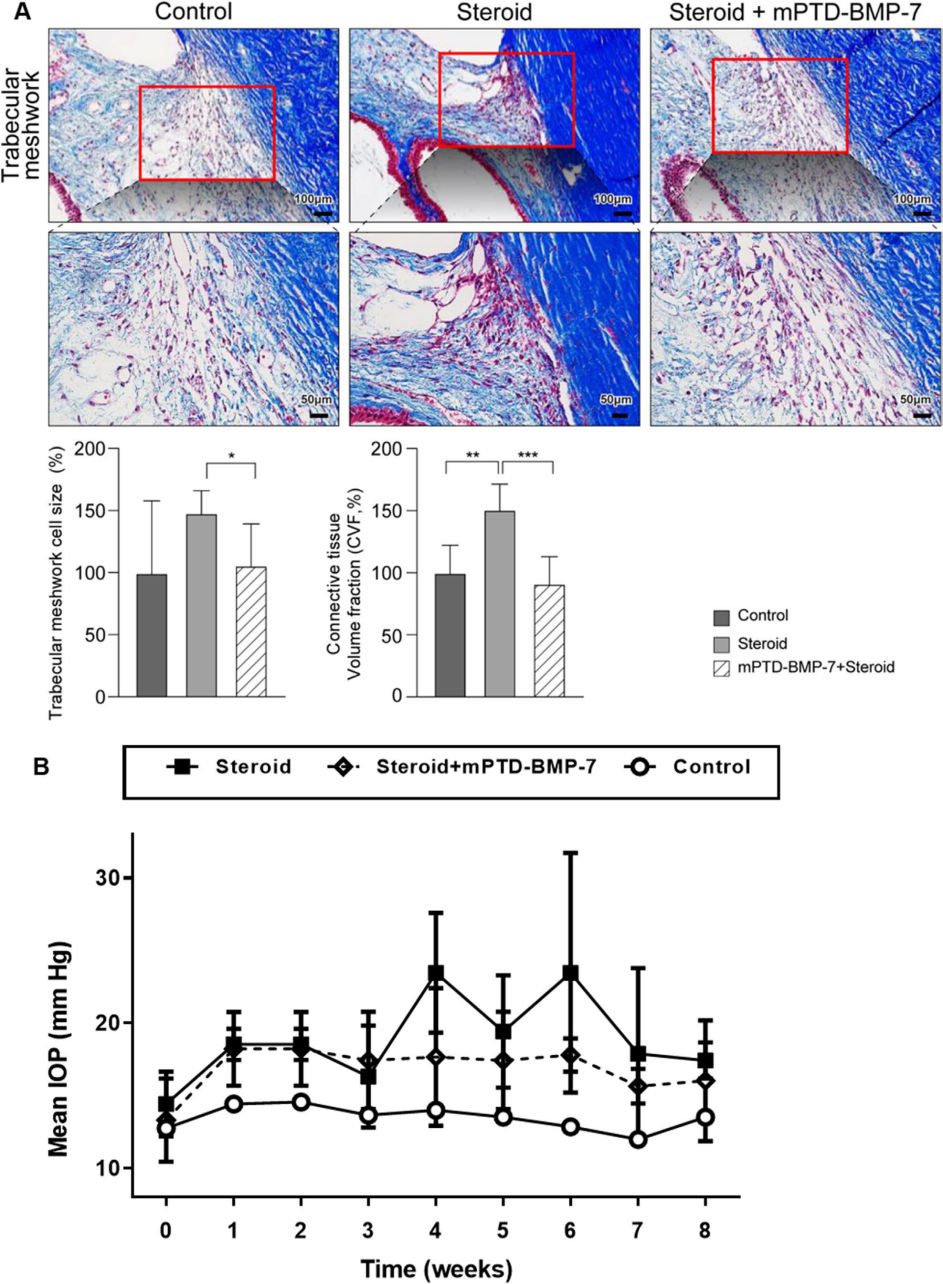
Effect of mPTD‐BMP‐7 on steroid‐induced changes in TM cell size and ECM volume. (A) Masson's trichrome staining of the TM in rabbits treated with dexamethasone (5 mg/mL) and mPTD‐BMP‐7 (20 ng/mL). Images were captured using a light microscope (upper row: Scale bar: 100 μm; magnification: 200×; lower row: Scale bar: 50 μm; magnification: 400×; the lower row images are magnified 2× from the upper row). Treatment with the steroid alone leads to increased cell size and connective tissue volume within the TM, whereas co‐treatment with mPTD‐BMP‐7 significantly mitigated these effects, restoring normal cell size and connective tissue volume. The data below confirm the quantitative analysis of changes observed in the images. (*, **, and *** indicate *p* < 0.05, *p* < 0.01, and *p* < 0.001, respectively). (B) Intraocular pressure (IOP) was measured over an 8‐week period following administration of either control (vehicle), steroid alone (dexamethasone 5 mg/mL), or steroid combined with mPTD‐BMP‐7 (dexamethasone 5 mg/mL and mPTD‐BMP‐7 20 ng/mL). Rabbits were used for IOP measurement, with three animals assigned to each group.

In the control group, the cytoplasm of TM cells (stained red) and connective tissue (stained blue) reflected intact structures within TM tissue. However, in the steroid‐treated group, there was a noticeable increase in the cytoplasmic area, indicating an increase in cell size, and substantial accumulation of connective tissue, suggesting an increase in the ECM. In the combination‐treated group, the cytoplasmic area was reduced relative to the steroid‐treated group, indicating that the increase in cell size was inhibited, and the amount of connective tissue was also reduced, reflecting decreased ECM accumulation. Quantitative analysis showed that cell size significantly increased in the steroid‐only group but returned to levels similar to those of the control group in the combination‐treated group. ECM quantification revealed a significant increase in ECM in the steroid‐treated group but returned to levels similar to those of the control group in the combination‐treated group. The result was quantified as CVF (Figure 4A) Also, IOP was measured over an 8‐week period following administration of either control (vehicle), steroid alone (dexamethasone 5 mg/mL), or steroid combined with mPTD‐BMP‐7 (dexamethasone 5 mg/mL and mPTD‐BMP‐7 20 ng/mL). Rabbits were used for IOP measurement, with three animals assigned to each group. The group treated with steroid combined with mPTD‐BMP‐7 exhibited a lower IOP increase compared to the steroid‐alone group (Figure 4B).

### 3.5 Identification of Differential mRNA Expression in HTMCs Treated With Steroid and mPTD‐BMP‐7

**FIGURE 5:**
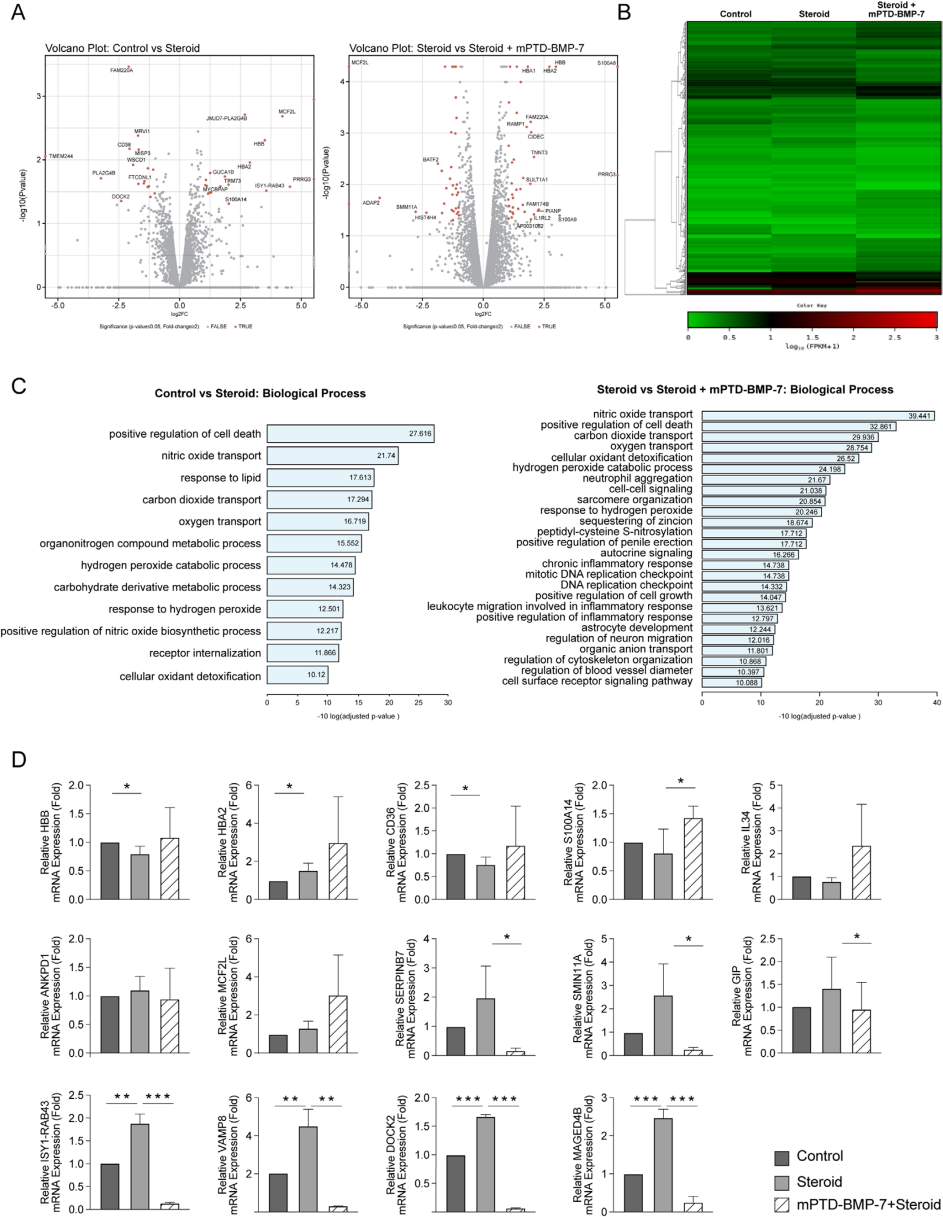
Gene expression analysis of HTMCs treated with dexamethasone and mPTD‐BMP‐7. (A) Volcano plots with log_2_ fold‐change (log_2_fc) versus −log_10_ (*p*‐value) representing the change in gene expression and its significance. These plots illustrate differential RNA expression between the control and dexamethasone co‐treated group (left plot) as well as between the dexamethasone‐treated and dexamethasone+mPTD‐BMP‐7‐treated groups (right plot). (B) Heatmap illustrating relative RNA expression in the control, dexamethasone, and dexamethasone+mPTD‐BMP‐7‐treated groups. (C) Gene Ontology (GO) analysis was conducted to identify the significantly enriched biological processes in the comparisons between the control versus dexamethasone and dexamethasone versus dexamethasone+mPTD‐BMP‐7. (D) RT‐qPCR confirmation and comparison of RNA‐sequencing results. The genes that showed the greatest change before and after dexamethasone+mPTD‐BMP‐7 treatment were VAMP8, DOCK2, ISY‐1‐RAB43, and MAGED4B, listed in the bottom row. (*, **, and *** indicate *p* < 0.05, *p* < 0.01, and *p* < 0.001, respectively).

The volcano plot (Figure 5A) highlights genes with statistically significant fold changes between the steroid‐only and steroid combined with BMP‐7 treatment groups, while the heat map (Figure 5B) visualizes the clustering of gene expression patterns across samples, emphasizing the distinct regulatory effects of these treatments. Pathway enrichment analysis was performed using Ingenuity Pathway Analysis and KEGG databases, revealing enriched biological processes and pathways (Benjamini–Hochberg FDR < 0.05). GO analysis (Figure 5C) further highlighted significant terms, with the largest clusters enriched in genes related to the regulation of cell death and nitric oxide (NO) transport.

Fourteen genes associated with related biological processes, identified through GO analysis (Figure 5C), were selected for RT‐qPCR validation. Among these, three genes—*SERPINB7*, *SMIN11A*, and *GIP*—exhibited significant changes specifically when steroid and BMP7 were administered together. Notably, four genes—*ISY1*‐*RAB43*, *VAMP8*, *DOCK2*, and *MAGED4B*—demonstrated consistent and significant alterations in expression across both the steroid‐only and steroid+BMP7 treatment groups, highlighting their potential key roles in response to both treatments (Figure 5D).

In addition to the genetic alterations observed following co‐treatment with steroids and BMP‐7, genes predominantly affected by steroids alone were categorized separately. Upon administration of steroids to HTMCs, 14 genes were upregulated while 16 genes were downregulated (Table 1).

### 3.6 Establishment of a Biological Interaction Network Based on Differentially Regulated mRNAs in HTMCs Co‐Treated With Steroid and mPTD‐BMP‐7

**FIGURE 6:**
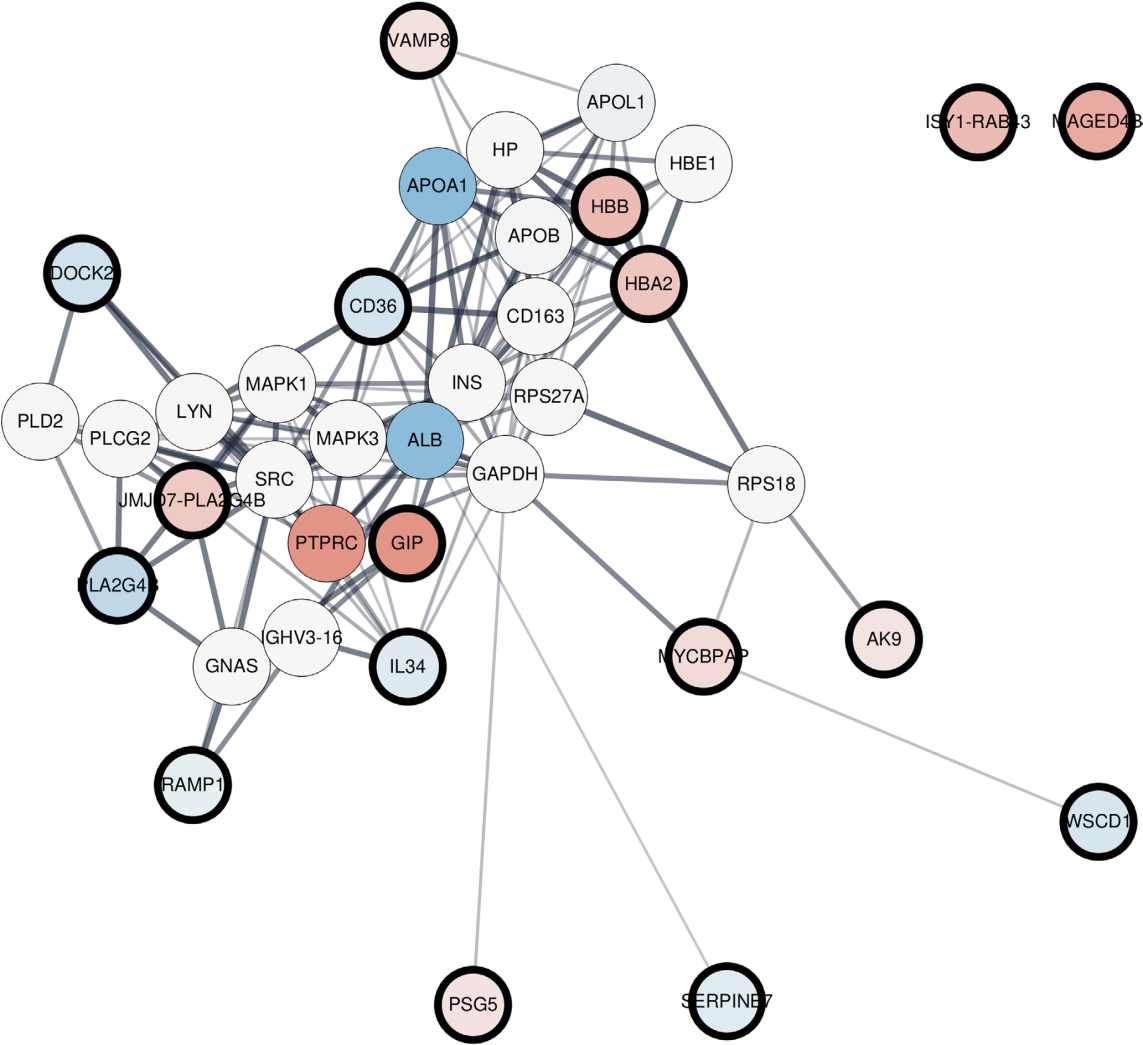
Protein–protein interaction (PPI) network of DEGs. A PPI network was constructed using STRING software to visualize the relationships among the 37 differentially expressed genes (DEGs) identified in this study. This analysis allowed for the identification of additional interactions, revealing how these genes interact within biological pathways. Among the four genes that demonstrated significance in the RT‐qPCR results from Figure 5D, DOCK2 and VAMP8 exhibited direct connections within this network, suggesting their potential key roles in the underlying biological processes. In contrast, ISY1‐RAB43 and MAGED4B did not show direct interactions with the other DEGs, suggesting a more isolated functional role.

As shown in Figure 5D, the expression of four genes was significantly upregulated or downregulated when treated with steroids alone or in combination with mPTD‐BMP‐7. Among these genes, *DOCK2* and *VAMP8* were found to be associated with the same biological process network. Additionally, *CD36*, which was significantly downregulated after treatment with steroids alone, and *SERPINB7*, which was significantly downregulated after combination treatment with both steroids and mPTD‐BMP‐7, were interconnected within the same network (Figure 6).

Using Cytoscape and its applications EnrichmentMap and AutoAnnotate, functional clustering was applied to the STRING‐generated network, producing 37 distinct DEGs. STRING analysis allowed us to further explore how these genes interact within biological pathways and processes. The node color corresponds to the log_2_ fold‐change indicated as log_2_fc. This visual representation highlights the extent of changes in certain genes in response to steroid or combined treatment, emphasizing the functional impact of these treatments on key biological processes.

## 4 Discussion

The ECM plays a crucial role in the structure and function of HTMCs, which are essential for regulating IOP by controlling the outflow of the aqueous humor from the eye [5]. The TM is a complex structure composed of beams and sheets of connective tissue, where ECM components such as collagen, elastin, fibronectin, F‐actin, and GAGs provide structural support, regulate cell behavior, and modulate aqueous humor flow [8, 18, 19].

In this study, significant changes in the ECM were observed at both the mRNA and protein levels in HTMCs. Notably, fibronectin exhibited substantial alterations at the genetic level, with mRNA expression patterns showing more pronounced differences on Day 3 of treatment compared to Day 2. These findings suggest a time‐dependent effect of the treatments on fibronectin production (Figure 2A). At the protein level, changes in the ECM and TGF‐β were observed, with the most pronounced changes occurring on Day 7. Specifically, there was a significant increase in fibronectin and TGF‐β2 levels when treated with steroid alone, whereas a notable decrease was observed when mPTD‐BMP‐7 was added. These findings highlight the potential role of mPTD‐BMP‐7 in modulating the effects of steroids on ECM protein synthesis. The pronounced changes in fibronectin and TGF‐β2 expression on Day 7 suggest that these effects became most significant over time, indicating a cumulative impact of the treatments (Figure 2B,C).

Fibronectin was the ECM component that exhibited the most significant increase in response to steroid treatment, accompanied by a notable rise in F‐actin levels, consistent with previous studies [20, 21]. This increase suggests potential densification of the extracellular space, with implications for cellular function and tissue structure (Figure 3A). The dynamic nature of ECM remodeling was further evidenced by the time‐dependent changes observed following treatment with steroids and mPTD‐BMP‐7. A rapid decrease in fibronectin and F‐actin levels occurred post‐treatment, with both returning to baseline levels by 6 h (Figure 3B). These findings indicate a significant reduction in ECM density after treatment, suggesting that the combination of steroids and mPTD‐BMP‐7 induces dynamic ECM changes that may influence tissue function.

TGF‐β and BMP‐7, both members of the TGF‐β superfamily, play crucial roles in various cellular processes, including tissue development, homeostasis, and fibrosis. Under normal conditions, TGF‐β and BMP‐7 signaling pathways interact and counterbalance each other to maintain tissue homeostasis. However, in fibrosis, this balance can be disrupted, leading to aberrant signaling that contributes to the pathological process. TGF‐β is well‐known for its pro‐fibrotic effects, promoting the production of ECM components and stimulating fibroblast activation, which can lead to excessive tissue scarring [8, 22, 23]. In contrast, BMP‐7 has been shown to have anti‐fibrotic properties, counteracting the effects of TGF‐β and promoting tissue regeneration and repair. Dysregulation in the balance between TGF‐β and BMP‐7 signaling, such as decreased BMP‐7 levels or increased TGF‐β signaling, can exacerbate fibrosis by promoting fibroblast activation, collagen deposition, and tissue remodeling.

Based on our finding that TGF‐β2 increases after steroid treatment and its known role in steroid‐induced glaucoma, we selected BMP‐7 as a candidate therapeutic antagonist. Although endogenous BMP‐7 levels were not measured in this study, future investigation of its expression and regulatory role in glaucomatous tissues may help further validate its therapeutic relevance [12].

SMAD proteins are intracellular signaling molecules that mediate TGF‐β family signaling from the cell surface to the nucleus, regulating gene expression [11, 24]. TGF‐β and BMP‐7 influence SMAD signaling in similar yet distinct ways: TGF‐β primarily activates Smad2 and Smad3, while BMP‐7 predominantly activates Smad1, Smad5, and Smad8 [25]. Although the precise mechanism through which BMP‐7 acts in HTMCs is not fully understood, it is thought that BMP‐7 and TGF‐β act within the same SMAD signaling pathway but at different regulatory points. This difference may allow BMP‐7 to selectively inhibit ECM production without disrupting TGF‐β's normal immune‐regulatory functions. Notably, mPTD‐BMP‐7 attenuated steroid‐induced p‐Smad3 activation, supporting its role in modulating the TGF‐β/Smad3 signaling pathway. Nevertheless, we acknowledge that comprehensive pathway profiling will be necessary in future studies to further elucidate its mechanistic effects (Figure S4).

Fuchshofer et al. observed changes in ECM‐related factors following the co‐administration of BMP‐7 and TGF‐β2 to HTMCs [12]. When TGF‐β2 was administered alone, there was an increase in CTGF, TSP‐1, fibronectin, collagen types IV and VI, and PAI‐1, which are ECM components. However, when co‐administered, the increase in these components was suppressed. No effects were observed when BMP‐7 was administered alone. This confirmed that BMP‐7 acts as an antagonist to TGF‐β2 signaling. However, this study diverges from previous research in several key aspects. Whereas prior studies focused primarily on the antagonistic relationship between BMP‐7 and TGF‐β, our investigation centered on BMP‐7's role in reducing the ECM accumulation increased by steroids. We aimed to explore the potential of BMP‐7 as a treatment for steroid‐induced glaucoma by examining its ability to counteract the increase in TGF‐β2 induced by steroid treatment. It is important to note that while BMP‐7 shows promise in preclinical studies, its efficacy and safety as a treatment for glaucoma, including steroid‐induced glaucoma, remain to be conclusively established in human clinical trials. BMP‐7 has been studied for its potential to attenuate fibrosis not only in the TM but also in other tissues, such as the kidneys, pancreas, and liver. Studies using mPTD‐BMP‐7 have demonstrated its increased efficacy in delivering BMP‐7's therapeutic effects to these tissues [11, 13, 26, 27, 28]. These findings suggest that mPTD‐BMP‐7, with its superior delivery and activity, may be a viable therapeutic agent across multiple clinical indications.

Gene expression analysis was conducted to elucidate the mechanism through which BMP‐7 inhibits ECM production and TGF‐β activity in HTMCs. A notable finding was the differential mRNA expression levels observed following steroid treatment alone versus in combination with mPTD‐BMP‐7. Specifically, the expression of genes, such as VAMP8, DOCK2, ISY1‐RAB43, and MAGED4B, increased with steroid treatment alone but decreased when combined with mPTD‐BMP‐7 (Figure 5D). VAMP8, primarily known for its role in vesicle trafficking and exocytosis, likely influences ECM remodeling by regulating the secretion of ECM components and remodeling enzymes [26, 29]. DOCK2, traditionally associated with immune cell migration and cytoskeletal reorganization, may intersect with BMP‐7 signaling pathways, thereby affecting ECM production in TM cells [30, 31]. ISY1‐RAB43, involved in intracellular vesicle trafficking, is hypothesized to affect cell size rather than directly influencing ECM synthesis, potentially altering secretion dynamics and cellular morphology [32, 33]. Lastly, MAGED4B, despite being less studied in ECM regulation, may modulate ECM synthesis through its effects on cellular signaling and gene expression, possibly interacting with established ECM‐related pathways such as TGF‐β signaling [34, 35]. Collectively, these genes may play significant roles in ECM regulation, thereby necessitating further investigation into their precise mechanisms.

The most significant genes, including VAMP8, DOCK2, ISY1‐RAB43, and MAGED4B, were validated through RT‐qPCR analysis. Given that VAMP8 and DOCK2 act within the same network (Figure 6), it is likely that these are related to the effects of steroids and BMP‐7 [36].

In this study, we investigated the potential of BMP‐7 as a therapeutic agent to inhibit ECM accumulation, a key factor in the development of steroid‐induced glaucoma. Our results demonstrated that BMP‐7 effectively suppresses ECM accumulation, indicating its potential for both the prevention and treatment of this condition. Additionally, we identified specific genes involved in the ECM reduction process, contributing to a deeper understanding of the molecular mechanisms underlying steroid‐induced glaucoma. This research suggests that BMP‐7 could serve as a valuable clinical therapy for managing the progression of this disease.

This study was conducted in vitro, and the potential in vivo toxicity of BMP‐7 remains to be tested. Further research is necessary to determine the appropriate dose and assess any potential side effects of BMP‐7 in humans.

## Author Contributions

E.W.K.: modeling, investigation, data analysis, and writing; J.‐O.C.: performed research, investigation, data analysis, and writing; M.K.C.: performed research, data analysis, and validation; J.‐S.L.: performed research, data collection, and validation; C.H.L.: data collection and investigation; J.E.U.: data collection and investigation; N.H.K.: contributed new reagents or analytic tools, data collection, and investigation; J.K.: contributed new reagents or analytic tools, data collection, and investigation; C.Y.K.: designed research, supervision, modeling, methodology, and investigation; W.C.: designed research, supervision, methodology, modeling, and validation. E.W.K. and J.‐O.C. contributed equally to this work as co‐first authors. W.C. and C.Y.K. are corresponding authors. The authors jointly interpreted the findings and wrote, revised, and approved the final manuscript.

## Conflicts of Interest

The authors declare no conflicts of interest.

## Supporting information


Data S1.

## Data Availability

The data sets used and/or analyzed in the current study are available from the corresponding author upon reasonable request.
