# Supplementary material for: Effects of Bone Morphogenetic Protein‐7 on Steroid‐Induced Extracellular Matrix Accumulation in Human Trabecular Meshwork Cells
Source: FASEB Bioadv. 2025 May 12;7(6):e70022. doi: 10.1096/fba.2025-00080 (PMC12147503; doi:10.1096/fba.2025-00080)
Supplement: Supplementary file 1 — Data S1. [file FBA2-7-e70022-s001.docx]

**Supplemental figure 1**

Characterization of primary human trabecular meshwork cells (HTMCs) by gene expression profiling.

**
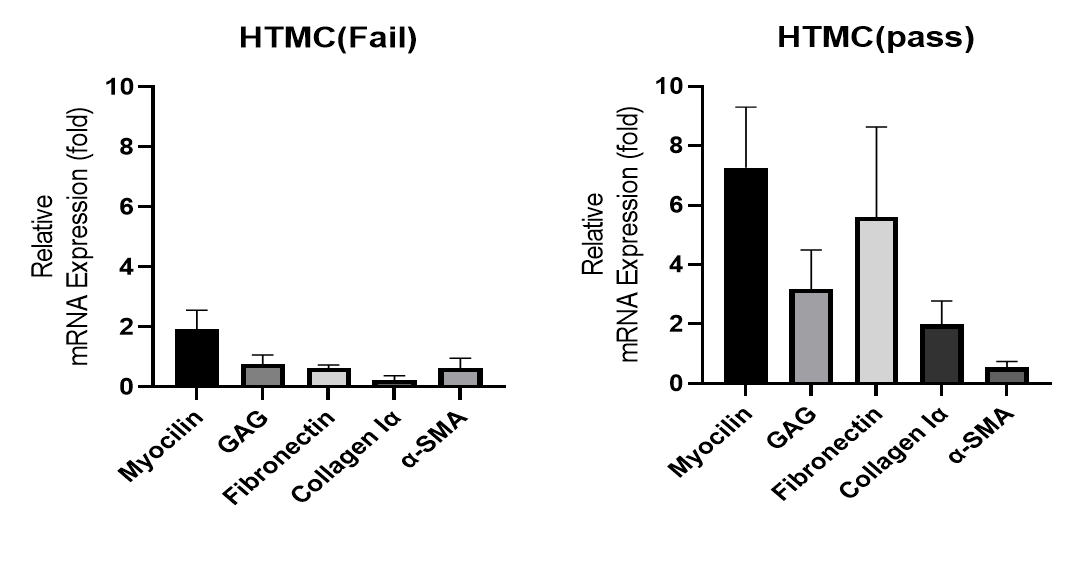
**

To authenticate the identity of the commercially obtained HTMCs, RT-PCR was performed to assess the expression of key trabecular meshwork cell markers after steroid treatment. MYOC (myocilin), GAG, FN1 (fibronectin), COL1A1 (collagen type I alpha 1), and ACTA2 (α-smooth muscle actin) was tested.

**Supplemental figure 2**

Testing the cell toxicity of *mPTD-BMP-7*with human trabecular meshwork cells

**
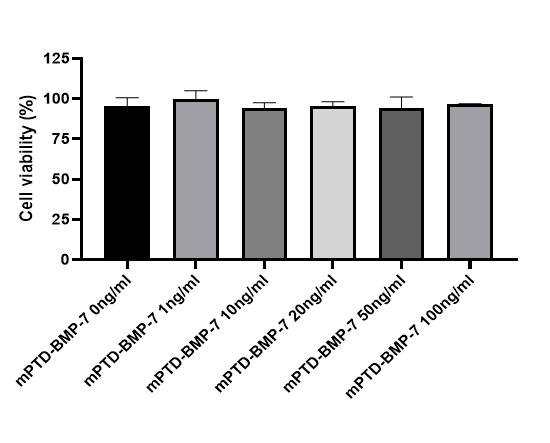
**

Primary human trabecular meshwork cells (HTMCs) were treated with 5, 10, 20,50 and 100 ng/mL of mPTD-BMP-7 for 24 hours, and cell viability was evaluated using the Cell Counting Kit-8 (CCK-8) assay. No significant decrease in cell viability was observed at any of the tested concentrations, indicating that mPTD-BMP-7 does not exert cytotoxic effects under these conditions.

**Supplemental figure 3**

Fluorescence intensity quantification of ECM components after steroid and mPTD-BMP-7 treatment in HTMCs by immunofluorescence staining


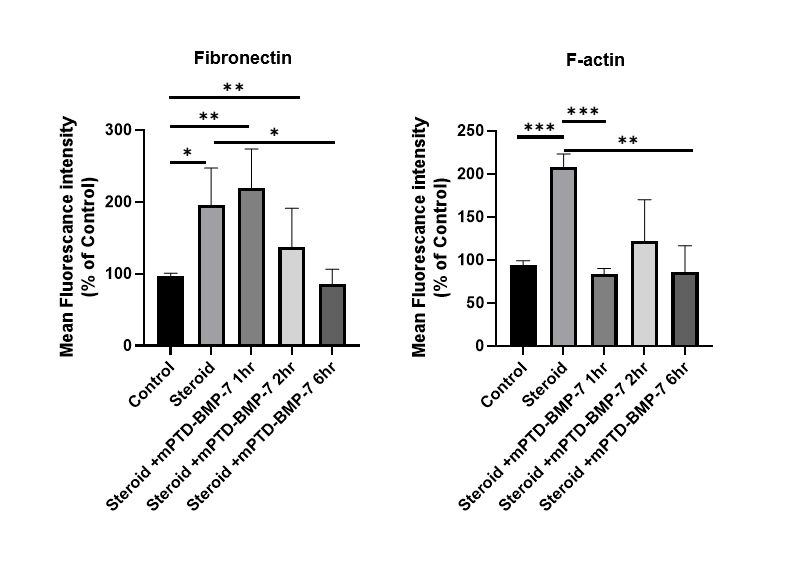


Fluorescence intensity quantification from three independent experiments were analyzed.

**Supplemental figure 4**

Effect of mPTD-BMP-7 on steroid-induced activation of the TGF-β/Smad3 pathway in HTMCs.

**
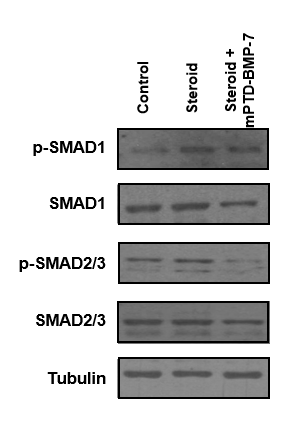
**

Western blot analysis of phosphorylated Smad3 (p-Smad3) and total Smad3 was performed in primary human trabecular meshwork cells (HTMCs) treated with DEX (100 nM) with or without co-treatment with mPTD-BMP-7 (20 ng/mL).

**Supplemental figure 5**

Molecular weight and size distribution of mPTD-BMP-7


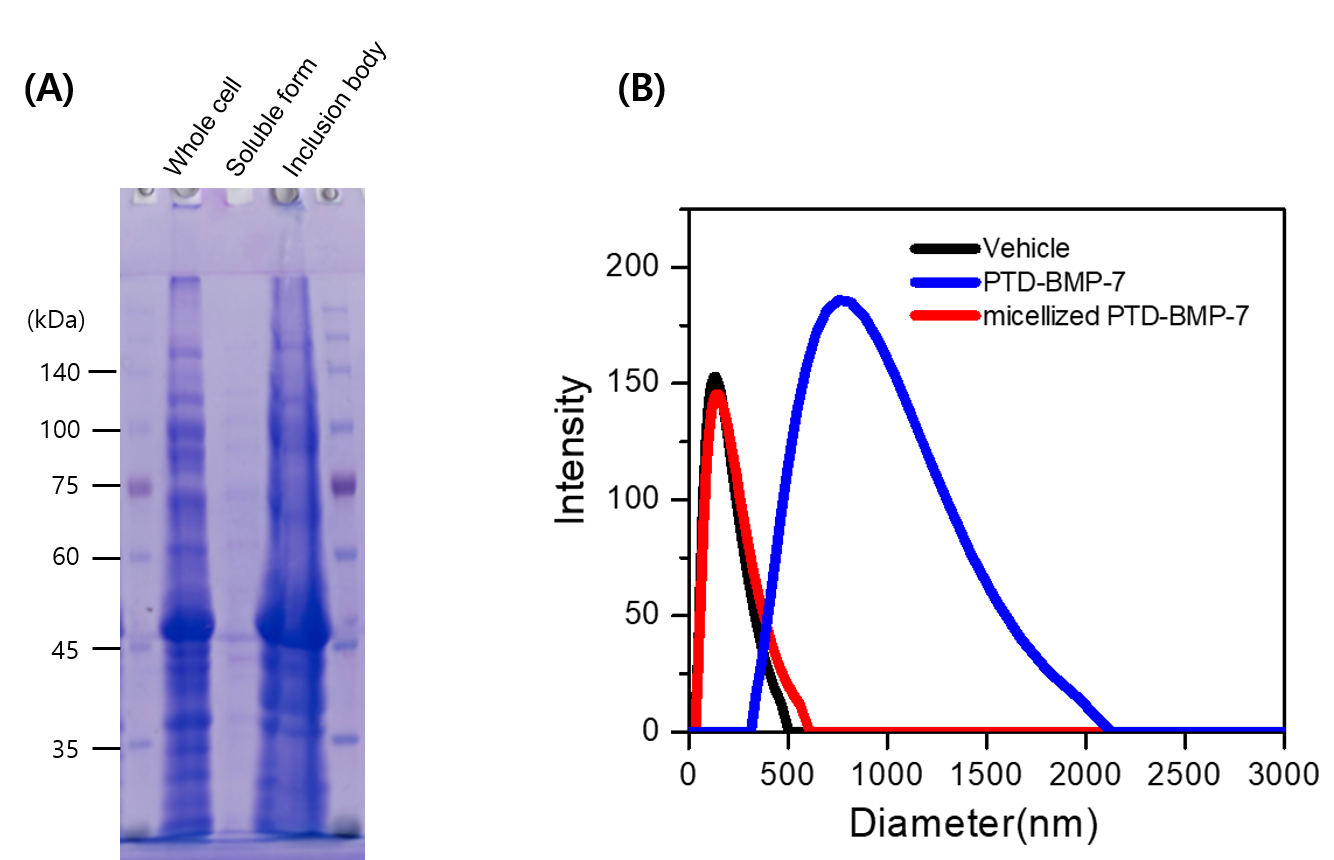


(A) Coomassie-stained gels reveal the molecular weight of mPTD-BMP-7 was shown at approximately 50 kDa location. (B) Dynamic light scattering (DLS). The particle size and distribution of the vehicle, non-mPTD-BMP-7, and mPTD-BMP-7 were assessed using a particle size analyzer (ELSZ-2000ZS, Otsuka Electronics) The effect of micellization on size distribution was tested using dynamic light scattering for the egg lecithin vehicle (black line), PTD-BMP-7 (blue line), and mPTD-BMP-7 (red line). Micellization resulted in a homogeneous and stable nanosized particle distribution.

**Supplemental TABLE 1** Antibodies used for western blotting.

| **Target protein** | **Company** | **Catalog number** |
| --- | --- | --- |
| Fibronectin | Santa Cruz Biotechnology | sc-8422 |
| GAG | Abcam | ab100970 |
| Collagen 1 | Santa Cruz Biotechnology | sc-293182 |
| α- SMA | Abcam | ab7817 |
| TGF-β | Cell Signaling Technology | 3711S |
| TGF-β 1 | Proteintech | 21898-1-AP |
| TGF-β 2 | Proteintech | 19999-1-AP |
| Alpha-Tubulin antibody | Sigma-Aldrich | T6199-100 |

**Supplemental TABLE 2** Primers used in quantitative reverse transcription polymerase chain reaction.

| **Gene** | **Sequence (5ʹ to 3ʹ)** |  | **Primers** |
| --- | --- | --- | --- |
| FIBRONECTIN | TGC GGC AGT TGT CAC AG |  | F |
|  | CCG TGG GCA ACT CTG TC |  | R |
| GAG | AGG AAG TAC CTG CCC CTC AT |  | F |
|  | CTC AGG ATT GGC GTT TTC AT |  | R |
| COLLAGEN 1 | CATGTTCAGCTTTGTGGACCT |  | F |
|  | GCAGCTGACTTCAGGGATGT |  | R |
| HBB | TGCGGGTCAAAGTGTACTTCC |  | F |
|  | ACTGCACGTCTAGGTTGAGTC |  | R |
| ANKRD1 | AGTAGAGGAACTGGTCACTGG |  | F |
|  | TGTTTCTCGCTTTTCCACTGTT |  | R |
| HBA2 | ACAAGCTTCGGGTGGACC |  | F |
|  | ACGGTATTTGGAGGTCAGCA |  | R |
| ISY1-RAB43 | TGGCAAGAGGAGAAAAGGAA |  | F |
|  | GCCTCCTCAATCTCTTGCTG |  | R |
| VAMP8 | TGTGCGGAACCTGCAAAGT |  | F |
|  | CTTCTGCGATGTCGTCTTGAA |  | R |
| MAGED4B | CAGCAAGATGAGGGTCCTGA |  | F |
|  | GCGCTTCATCCCCGATATTC |  | R |
| DOCK2 | AGAAATGTCAAAAGACCAGCCA |  | F |
|  | TATGACCGTTTGCTTGTTGGG |  | R |
| CD36 | GGCTGTGACCGGAACTGTG |  | F |
|  | AGGTCTCCAACTGGCATTAGAA |  | R |
| MCF2L | TGTTGTTATCGACAGACGAAGAG |  | F |
|  | AAGCGAGATGGACGAAGGATG |  | R |
| GIP | AAGTGGACATGGACAAGCTCC |  | F |
|  | CCTCCGACTTGAACACCTCC |  | R |
| IL34 | CCTGGCTGCGCTATCTTGG |  | F |
|  | AGTGTTTCATGTACTGAAGTCGG |  | R |
| S100A14 | GAGACGCTGACCCCTTCTG |  | F |
|  | CTTGGCCGCTTCTCCAATCA |  | R |
| SERPINB7 | AAATGCAGAGTTTTGCTTCAACC |  | F |
|  | GAAGAGTTTCCATATCCTGAGGC |  | R |
| SMIM11A (C21orf51) | AATTGGAAGGTCCTAACTGGGA |  | F |
|  | CTTGTCGGCAAAGTGGTGG |  | R |
| GAPDH | CAACGGATTTGGTCGTATTGG |  | F |
|  | GGCAACAATATCCACTTTACCAGAGT |  | R |
